# Funds allocation in NPOs: the role of administrative cost ratios

**DOI:** 10.1007/s10100-017-0512-9

**Published:** 2017-12-27

**Authors:** Christian Burkart, Tina Wakolbinger, Fuminori Toyasaki

**Affiliations:** 1 Institute for Transport and Logistics Management, WU (Vienna University of Economics and Business), Welthandelsplatz 1, 1020 Vienna, Austria; 2 School of Administrative Studies, York University, 4700 Keele Street, Toronto, ON M3J 1P3 Canada

**Keywords:** Funds allocation, Performance measures, Analytic model, Utility function

## Abstract

Performance measurement of Non-Profit Organizations (NPOs) is of increasing importance for aid agencies, policy-makers and donors. A widely used benchmark for measuring the efficiency of NPOs is the overhead cost ratio, consisting of the total money spent on administration and fundraising relative to the budget. Donors generally favor a lower overhead cost ratio as it ensures that more money directly reaches beneficiaries. Unlike fundraising expenses, administrative costs do not contribute to advertising the actions of an NPO even though they account for a significant proportion of overhead cost. Reducing administrative expenses is a logical consequence from a financial viewpoint, but might negatively affect NPOs through the resulting administrative capacities. This phenomenon is known as “Nonprofit Starvation Cycle”. This work provides an analytical framework for analyzing NPO decision making concerning administrative costs. The paper provides answers to important research questions on the optimal level of administrative spending, the influencing factors and the effects of available information on NPOs. The research shows that focusing on financial performance measurements can result in reduced utility created for NPOs. Less transparency often leads to increased utility for NPOs, but more transparency can increase NPOs’ utility if the information available exceeds a certain threshold. Fluctuating donations are challenging for NPOs’ planning and may impact administrative capacities negatively.

## Introduction

Private donors face an ever growing number of organizations asking for their support and have to decide on whom to donate to (Kovács and Spens 2007). Donors cannot readily obtain information about whether organizations use their funds in the way the donors intend or do so in the most efficient way. Benchmarks are a tool for assessing the performance of an NPO, well suited to support the decision making process of the donor. An easily accessible and widely used benchmark is the ratio overhead costs relative to total expenses, since these numbers can be extracted from publicly available accounting information. Overhead costs consist of administrative and fundraising costs (Bowman 2006), they comprise all expenses that are not related to programs, and therefore, do not directly create impact. These costs include costs for staff at headquarters, office supplies, marketing and rent. However, no standardized definition of administrative costs exists. Often, administrative costs are labeled as part of indirect costs, which are used in a supportive way to facilitate the use of the direct costs, accounting for project expenses. The United Nations Central Emergency Response Fund Secretariat (2012) describes administrative costs as costs for “[...] recruitment and servicing of staff, consultants and fellowships, procurement and contracting (usually centralized), budget preparation and control, financial operations, accounts, reporting, and auditing”. As Ortiz (2001) shows in a study conducted for, among others, USAID, there is no single standardized way to attribute costs to their respective categories, but three groups of costs have been defined: General management costs for all activities, research and development costs and support services, including costs for premises, IT, finance costs and administrative, personnel and training costs. Inconsistencies in definition have been criticized by the United States Government Accountability Office (2010).

At the same time that donors decide on whom to donate to, NPOs have to balance the allocation of funds in order to achieve goals and sustain the future inflow of donations, enabling the creation of future impact. On the one hand, donations cannot be diverted from the organizations’ intended usage according to the non-distribution constraint (Hansmann 1987), which states that while NPOs are allowed to make profits, they are not allowed to distribute net earnings but have to use them for the organizations’ purposes. On the other hand, donations can be even bound to a certain type of usage, like the response to a specific disaster, which has become known as “earmarking” in the literature (Toyasaki and Wakolbinger 2014 or Bhattacharya et al. 2014).

Therefore, NPOs face a basic decision of using the received, non-earmarked, donations either for the programs they conduct, for increased fundraising activities, or for increased administrative capacities. The direct link between such financial decisions in general, operations of an NPO and its efficiency has been described by Wakolbinger and Toyasaki (2011) for the case of funding systems. While increased program expenses can increase the impact created by NPOs’ operations and attract even more donations, the effect of increased fundraising expenses is ambiguous. Okten and Weisbrod (2000) describe two countervailing effects connected to increases in fundraising expenses: On the one hand, fundraising has a cost effect, reducing the efficiency of NPO operations through additional, not impact creating, expenses, but on the other hand, the advertisement effect attracts higher donations. Lastly, following this line of arguments, administrative costs, which consist of costs that cannot be clearly attributed to specific programs or fundraising, provide only a cost effect without advertisement, thus no positive effect on the sum of donations.

From this viewpoint, the development of the “Nonprofit Starvation Cycle” (Lecy and Searing 2015) can be considered as a foreseeable development: Triggered by donor expectation concerning ‘acceptable’ overhead ratios and NPO rating organizations publishing benchmarks based on overhead ratios, donors reward low ratios by donating more to seemingly ‘efficient’ NPOs. This lures NPOs into decreasing the ratio of overhead to gain a competitive advantage in donation ‘markets’, further elevating donor pressure by increasingly unrealistic expectations. At some point, overhead costs can only be decreased by choosing between reducing necessary administrative capacities or engaging in misreporting on overhead used as a misleading tactic to succeed in this price war turned into a race to the bottom (Lecy and Searing 2015).

While many authors have described this development under different names [“Nonprofit starvation cycle” (Wing and Hager 2004), “Evaluability bias” (Caviola et al. 2014) or “New bottom-line movement” (Frumkin and Kim 2001)]. Some even suggest engaging in this creative accounting by using large donations from major philanthropists to cover overhead costs and advertising to other potential donors that their donations will go in full towards the programs to avoid this “overhead aversion” (Gneezy et al. 2014). This behavior is supported by findings that the quality of the reported ratios has limited impact on donations (Yetman and Yetman 2013).

The reliance on overhead cost ratios as quality indicators of NPO operations has been unanimously identified as detrimental to the NPO sector. Studies find no correlation between low overhead expenses and high efficiency of NPOs (Caviola et al. 2014). Infrastructure investments are a necessity for making good use of resources (Bhattacharya et al. 2014). Lack of marginal data concerning costs render overhead ratios useless as decision support indicators (Tinkelman 2006).

This article will focus on the role of administrative costs as part of overhead costs. Although the majority of overhead costs consist of administrative expenses (Lecy and Searing 2015), much work about the impact of fundraising expenses on the behavior of NPOs was conducted. Relatively little research has been conducted on the impact of administrative costs in NPO operations, as mentioned by Tinkelman and Mankaney (2007) and Aldashev and Verdier (2010). Based on a compound utility function for NPOs, the optimal level of administrative cost ratios is investigated through the development of an analytical model of NPO actions. Hence, the following questions will be investigated in this paper:

- What is the ‘optimal’ level of the administrative cost ratio for an NPO?
- How do changing donation levels, fundraising expenses, efficiency gains from administrative spending and donor reaction to overhead information affect optimal administrative expenses?
- Is the recent trend for more information about overhead costs provided to donors beneficial for NPOs?

Our research shows that the focus on financial performance measurements in NPOs and resulting reduced administration cost ratios can reduce the utility created for NPOs. Less transparency can lead to increased utility for NPOs. Fluctuating donations pose a challenge to NPOs’ planning and can result in negative impact on administrative capacities.

The remainder of this paper is structured as follows: Sect. 2 presents models and a brief description of the relevant parameters, followed by Sect. 3 introducing the numerical studies conducted. This includes optimality and sensitivity analysis and an extension of the model, which contains the degree of transparency concerning financial information. Section 4 concludes the paper, proposing future research opportunities and pointing out managerial insights.

## Model

**Fig. 1:**
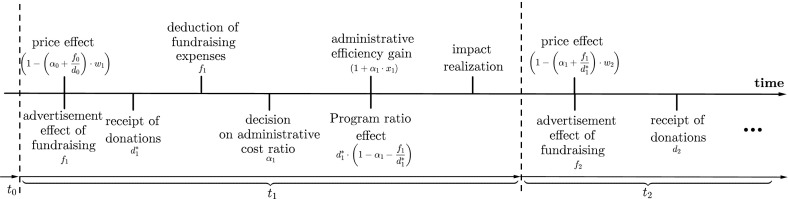
Sequence of events

The model proposed for the analysis of the effects and impact of administrative cost ratios takes the financial decision-making of one single NPO into account. Generally speaking, the budget acquisition process of an NPO follows a common pattern, which can be seen in Fig. 1.

First, donations are solicited with support of fundraising. The received donation sum $d_1^*$ depends on the base donation amount $d_1$ for this specific time period and the NPO’s previous overhead cost performance, potentially increasing or decreasing this base donation amount. This ‘price effect’ will be described later in more detail. The base donations describe the donation potential that could be solicited by the NPO in question, including all factors that are not directly related to administrative expenses like recurrent donations made by an established donor base, the reputation of the NPO, the appeal of current programs, donor willingness to donate, the advertisement effect of fundraising expenses attracting new donors and many more. After receipt of donations, fundraising expenses $f_1$ have to be covered. Then the remaining funds are allocated to their different forms of usage, either for administration or directly for the programs.

This paper enters this process at this decision-making stage of funds allocation. The NPO decides upon the level of administrative costs relative to total budget and maximizes the utility created. The effects of the decision are twofold, influencing the utility in two consecutive periods (accounting years): firstly they determine the effect on the impact generated by the NPO for the beneficiaries in the present period through improving the efficiency of the NPO if administrative spending corresponds to the operational needs for administrative support and through changes in the program ratio. This is the ratio of funds available spent directly on the programs, which is reduced if administrative expenses are increased. Both effects influence the realized impact in the current period. Secondly the future budget is negatively influenced by the level of the overhead cost ratio. While the fundraising expenses and their resulting advertisement effect increasing future donations are not investigated in this paper in detail,^1^ the negative impact of donor awareness about higher overhead ratios on future donations (price effect) is considered in detail.

The decision on the design of the objective function is of central importance for the proposed model. A frequently employed approach is using the available budget as a measure for utility, which is to be maximized. This budget maximizing approach does not consider all aspects of the environment an NPO acts in. Martinez et al. (2011) and Aldashev and Verdier (2010) use the utility of different groups with potentially diverging objectives, including groups like beneficiaries and donors. It can be expected that NPOs take these stakeholders into account when planning their actions, especially donors are sometimes regarded as the ‘real’ customers in a humanitarian relief situation, which is due to the dependence on and competition for funding (Balcik et al. 2010).

In this section, we introduce an NPO utility function, which is a two-stage compound utility function. Following Steinberg (1986), the utility of an NPO is associated herein with the success in financially sustaining their operations through maximizing current and future impact. Additionally, the objective of the beneficiaries is taken into account via maximizing the current impact of the NPOs’ programs, which is assumed to be identical to the goal of the donors, as suggested by Weisbrod and Dominguez (1986). This section describes first the required parameters and variables, then the utility function is described in more detail.

### Decision variable and parameters

The proposed model is based on the analysis of one single decision variable, $\alpha _1$, the ratio of the total budget used for administrative purposes and employs a variety of parameters. As the utility function is a compound utility function over 2 periods, describing current impact (first period) and future impact (second period) maximizing goals, some parameters can be associated specifically with one period while others are used throughout the function. An overview of the employed parameters and auxiliary variables (including parameters for model extensions) and their limits can be found in Table 1.

**Table 1:** Notation and assumptions

Decision variable	
$\alpha _1$	Administrative cost ratio
*Parameters current period* $t_1$	
*a*	Utility function weight parameter
$\alpha _0$	Administrative expense ratio in previous period
$d_1^*$	Realized donations current period
$d_0^*$	Realized donations previous period
$d_1$	Base donations current period
$f_0$	Fundraising expenses in previous period
$f_1$	Fundraising expenses current period
$w_1$	Price effect of overhead of previous period
$x_1$	Efficiency gain parameter
*Parameters future period* $t_2$	
$\alpha _2$	Administrative expense ratio in future period
*b*	Discount factor
$d_2$	Base donations future period
$d_2^*$	Realized donations future period
$f_2$	Fundraising expenses in future period
$g_2$	Future period efficiency gain effect
$i_2$	Information parameter
$p_2$	Future program ratio
$w_2$	Price effect of overhead
$x_2$	Future efficiency gain parameter
*Auxiliary variables*	
$d_1^*$	$d_1 \cdot (1- (\alpha _0 + \frac{f_0}{d_0}) \cdot w_1)$
$g_2$	$1+\alpha _{2} \cdot x_2$
$p_2$	$1-\alpha _2 - \frac{f_2}{d_2}$
*Assumptions*	
$1> a > 0$	Positive weight values
$1 \ge b \ge 0$	Depreciation factor limits
$d_{0,1,2}^*>0$ and $d_{0,1,2} > 0$	Non-negativity of donations
$1 \ge i_2 \ge 0$	Information level limits
$f_{0,1,2} \ge 0$	Non-negativity of fundraising expenses
$$ w_{1,2} > 0$$	Price effect limits
$x_{1,2} > 0$	Positive efficiency gain parameter
$(p_2, g_2) \ge 0$	Non-negativity of unmodeled effects

### A basic model

As can be seen in Model 1a, the utility function consists of two components, current impact and future impact maximizing parts that are weighted by *a* and $1-a$ respectively. To limit the influence of the parts to positive values, the weight parameter is limited to $1> a > 0$. While, for calculations in this paper a value of $a=0.5$ is assumed to depict an NPO indifferent between the two components, a higher value of *a* would represent a stronger focus on current impact generation while a smaller value of *a* would represent stronger focus on sustaining future operations. These two parts will be described in detail in the following sections.

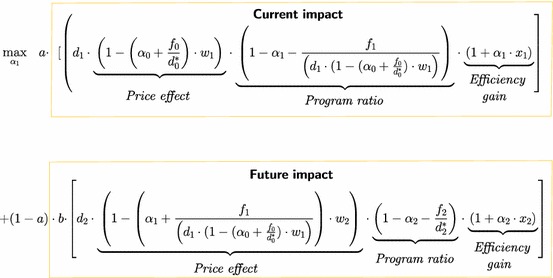



**Model 1a:** Compound utility function

Both periods contain the effects on the impact created resulting from

- the price effect (i.e. the effect of available overhead cost information on the donation behavior)
- the program ratio (i.e. the residual budget spent on programs after deducting overhead costs)
- the efficiency gain (i.e. the increase in impact resulting from the more efficient use of program expenses due to increased administrative capacities)

To make this long formulation a little easier to grasp, all effects leaping over from previous periods or leaping over to future periods which do not directly depend on our decision variable $\alpha _1$ are abbreviated by auxiliary variables. These auxiliary variables contain only parameters and can therefore be treated as single parameters as well. We continue by describing the different effects of the current and future period in the abbreviated Model 1b which is used in the remainder of the paper and end this description with an explanation of the auxiliary variables.

#### Period 1: current impact

In the current period, the donations that have been collected, $d_1^*$, are used as a budget to create impact. The first period accounts for the current impact maximizing part of the utility function. For the calculations, a donation sum of $\$$550,000 is assumed, as this is part of the range of NPO size where overhead expense ratios play an important role, as Lecy and Searing (2015) show. This period combines the program ratio, the ratio of donations available for programs, with the efficiency gain effect of increased administrative expense ratios.

*Program ratio*

Out of this available budget $$d_1^*$$, the ratio that can be used for projects, and thus impact, is the residual ratio after subtracting the ratio for administrative tasks and the expenses for fundraising, therefore the multiplication with $$1-\alpha _1-\frac{f_1}{d_1^*}$$ is required. If either the ratio used for administration or for fundraising are increased, the money effectively spent on programs is reduced accordingly. For the calculations contained in this paper, a value of three percent for $$\frac{f_1}{d_1^*}$$ is considered following the recent contribution of Lecy and Searing (2015), resulting in $$f_1$$=16,500$.

*Efficiency gain*

Since the impact achieved by the program budget depends not only on the amount of money spent on programs, but also on the efficiency of the administration of the programs, the efficiency gains realized by the administrative expenses are accounted for by including the term $1+\alpha _1 \cdot x_1$. Hence, $x_1$ is a parameter describing the linear efficiency gains depending on the level of administrative cost relative to budget, $\alpha _1$. Any increase in $x_1$ increases the positive effect of administrative expenses on the fulfillment of the NPO goals through the programs, increasing the impact created at stable program expenses. For calculations, a value of $x_1=2$ is assumed. The notion that decreased administrative spending reduces the capacity of the NPO is brought forward by many authors discussing the Non-Profit Starvation Cycle, most notably Lecy and Searing (2015) and Wing and Hager (2004). Our model assumes that efficiency gains are often not cumulative, i.e. previous administrative expenses do not guarantee higher efficiency in the current period. Administrative expenses for staff salaries, rent or office supplies constitute a substantial proportion of administrative expenses, but have no effect on the efficiency of future periods. On the other hand, if more long-term investments are made, their costs are attributed often to the period in which they are used. Examples for this are expenses for software licenses for a new accounting system, which would be due in each period, or depreciable costs of real estate resulting from investments into office buildings. Therefore, efficiency gains have no multiperiod effects but depend on the administrative expenses of each separate period.

#### Period 2: future impact

This period includes the impact of the future budget situation on the impact realized and, thus, the decision-making process of the NPO. The discounted future donations depend on the (unmodeled) advertisement effect of fundraising expenses and the impact of the level of overhead expenses (price effect) on the donations of the next period. Additionally, the program ratio and efficiency gain are included through auxiliary variables $g_2$ (efficiency gain in the future) and $p_2$ (program ratio effect of the future period).

For developing a future impact-related utility measure, a well developed approach is followed, describing the interaction of administrative cost ratios and donation level. Therefore, in analogy to the “price of giving” (Weisbrod and Dominguez 1986) we use a metric for the influence of overhead cost increasing this cost [(as used for example in Bowman (2006)], and fundraising or admin cost (Greenlee and Brown 1999) in more detailed studies and frequently applied since then, for example Khanna and Sandler (2000). This follows the idea of a ‘market’ for donations, where more ’expensive’ NPOs receive fewer donations due to their smaller fraction of donations spent on programs.

The terms used for the future, or second, period in the utility function describe the aim of the NPO to maximize its future impact. A donor can judge the NPO according to the data available on overhead cost. Assuming that there is a maximal donation sum that can be solicited, this would be reduced relative to the ratios of overhead $\alpha _1 +\frac{f_1}{d_1^*}$, but corrected for the cost effect on donations $w_2$ and, since the resulting donations will not be available in this period, a discount factor *b*.

*Discount factor*

As the future budget does not become available in the current, or first, period, monetary values have to be discounted according to the preference of the NPO. As *b* describes the discount factor, a low value would indicate little utility gained from future donations, while a higher value would emphasize the benefits of future donations, sustaining operations in the periods to come. A high preference for future donations could be especially prevalent in a period, in which a lot of funding is available for a very prominent cause, like in the immediate aftermath of an earthquake. In such a situation, it is important to sustain financial flows even after the time of peak donations resulting from peak media attention, as the response to such disasters continues much longer than media attention. For the calculations included in this paper, the average global inflation rate in consumer prices of currently 4.7% is used, as provided by the International Monetary Fund in its World Economic Outlook (International Monetary Fund 2017). A realistic setting of one year periods results therefore in $b= \frac{1}{(1+0.047)}$. Therefore, *b* is assumed to be 0.9551.

*Price effect*

The next multiplicative term influencing future donations includes fundraising and administrative expenses as part of a ‘price effect’ inspired by the “price of giving” of Weisbrod and Dominguez (1986), including administrative ratio $\alpha _1$ and fundraising expense ratio $\frac{f_1}{d_1^*}$ increasing the price of giving for the donors, expressed by $p=\frac{1}{1-( \alpha _1 +\frac{f_1}{d_1^*})}$. This is multiplied by the effect $w_2$ of this overhead cost ratio on the donation sum, constituting the price effect. Borrowing again from the literature, Peloza and Steel (2005) found for more than 50 regression analyses an average value of $-1.44$ for donation elasticity of overhead cost.

For the calculations provided in this paper, the value of $w_2$ is calculated using a donation function with constant price elasticity of demand of $-1.44$. The donation function *D* consists of the price of giving *p* multiplied with an price effect parameter $w_2$, hence $D=p \cdot w_2$. Given the empirical data from Peloza and Steel (2005), a donation function with constant elasticity can be formulated like $D = p^{-1.44}$. This donation function can be rearranged to resemble the format of $D=p \cdot w_2$ through the expansion of $D= p \cdot p^{-1.44 - 1}$, with $p^{-1.44 - 1}$ behaving like $w_2$. Now, the average values for the reasonable range of the overhead ratio of $0.03 \le (\alpha _1 +\frac{f_1}{d_1^*}) \le 0.5$, or, in terms of price of giving *p*, $1.03093 \le p \le 2$, of $w_2$ are calculated. This leads to a value of $w_2 = 0.37204$, which will be used in the following calculations.

#### Auxiliary variables

In order to arrive at total impact created in both current and future period, the effects leaping over from previous periods and leaping into future periods have to be taken into account as well. In the current period, the price effect of the previous period is acknowledged by including it implicitly in the realized donation sum $d_1^*$. This budget $d_1^*$ is an auxiliary variable for $d_1 \cdot (1- (\alpha _0 + \frac{f_0}{d_0^*}) \cdot w_1)$, describing the changes to the base donations $d_1$ through donors considering previous overhead ratios.

The future impact relies also on effects which are included through auxiliary variables, $p_2$ for the effect of future program ratio and $g_2$ for the efficiency gain effect. The future program ratio can be formulated as $p_2 = 1-\alpha _2 - \frac{f_2}{d_2^*}$, depending on future administrative cost ratios and fundraising expenses relative to future realized donations. For calculations, we assume $\alpha _2$ to be 15.3% and $\frac{f_2}{d_2^*}=3$%, as identified as average values by Lecy and Searing (2015). Therefore, $p_2$ equals 0.817.

The future efficiency gain is described by $g_2 = 1+\alpha _{2} \cdot x_2$, depending on the future administrative ratio $\alpha _2$ and future efficiency gain parameter $x_2$. Using the same level of $\alpha _2$ as in the calculation for $p_2$ and assuming $x_2 = x_1$ results in $g_2 = 1.306$, which is used for further calculations.

The complete, abbreviated model can be seen in Model 1b.

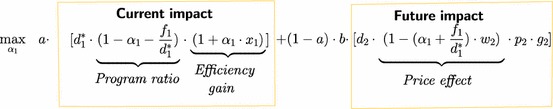



**Model 1b:** Compound utility function (auxiliary variables)

subject to

1 $$\begin{aligned} \alpha _1 +\frac{f_{1}}{d_1^*}&\le 1 \end{aligned}$$

2 $$\begin{aligned} \alpha _1&\ge 0 \end{aligned}$$

## Numerical studies

After introducing the intuition of the model, in this section the model is analyzed in detail. First, optimality and sensitivity analysis is conducted, which is followed by the investigation of the implication of the information about overhead cost available to donors.

### Optimality and sensitivity analysis

Since the second derivative of the objective function is found to be $f''(U)=-2\cdot a \cdot x_1 \cdot d_1^*$, it becomes clear that the function is necessarily concave, as $a, x_1, d_1^* >0$, thus any extreme point found will be a global maximum. The assumptions that donations $d_1^*$ are a positive amount and the efficiency gain factor $x_1$ is positive is very reasonable, as benefits of increased administrative expenditure on administrative efficiency can be assumed. A positive value of *a* is a reasonable assumption, as it translates into at least some interest by the NPO into optimizing current impact generation. However, the case of $a=0$ could occur. In this special case, the utility function is reduced to the impact maximizing part in period 2 of $d_2 \cdot b \cdot (1-( \alpha _1 +\frac{f_1}{d_1^*}) \cdot w_2 \cdot p_2 \cdot g_2)$. In such a case, the optimal level of $\alpha _1^*$ would be equal to 0. As the utility function is designed to include the utility of different stakeholders, this special case will not be dealt with in the remainder of the paper.

The optimal level of administrative cost ratio $\alpha _1^*$ is given by Eq. 3:

3 $$\begin{aligned} \alpha _1^*=\frac{1}{2}- \frac{1}{2 \cdot x_1}- \frac{f_1}{2 \cdot d_1^*}- \frac{(1- a) \cdot b \cdot d_2 \cdot p_2 \cdot g_2 \cdot w_2 }{2 \cdot a \cdot d_1^* \cdot x_1} \nonumber \\ \text {under the condition that constraints (1) and (2) are fulfilled} \end{aligned}$$

The level of utility created at $\alpha _1^*$, $U^*$, can be easily calculated using the equations obtained:

4 $$\begin{aligned} U^*&=\frac{a \cdot d_1^* - (a-1) \cdot b \cdot d_2 \cdot g_2 \cdot p_2 \cdot w_2)^2}{4 \cdot a \cdot d_1^* \cdot x_1} + \frac{a \cdot (d_1^* - f_1)^2 \cdot x_1}{4 \cdot d_1^*} \nonumber \\&\quad +\, \frac{a \cdot d_1^* \cdot (d_1^* -f_1) - 2 \cdot (a-1) \cdot b \cdot d_1^* \cdot d_2 \cdot g_2 \cdot p_2 + (a-1) \cdot b \cdot d_2 \cdot (d_1^*+f_1) \cdot g_2 \cdot p_2 \cdot w_2}{2 \cdot d_1^*} \end{aligned}$$

#### Proposition 1

The optimal level of the administrative cost ratio $\alpha _1^*$ is bounded by $\alpha _1^* < 0.5$

Proposition 1 indicates, that the highest possible value for the optimal level of administrative cost ratio $\alpha _1^*$ is 0.5, which can be derived from Eq. 3. This high value can only be approximated if $f_1=0$ and $x_1$ takes a very large value. This can be explained by the two countervailing effects inherent in the utility function, with negative and positive influences of increased administrative spending in the first period (reduced program ratio but increased efficiency) and negative impact in the second (increased price of giving).

After deriving the optimal administrative cost level we explore the sensitivity of these partial derivatives for the parameters to describe the changes in the optimal level of $\alpha _1^*$ for changes in the parameters as can be seen in Table 2.

The weight of the utility function concerning the periods *a* has a positive effect on the level of $\alpha _1^*$, which is due to the fact that the weight *a* is the weight of the current impact part, while $1-a$ describes the weight of the future impact part. If *a* increases, more of the utility created comes from the current period, increasing the importance of administrative efficiency. However, this effect is decreasing in *a*. This result indicates that organizations that focus more strongly on future budget and impact maximization than current impact tend to have a lower $\alpha _1^*$ than NPOs focusing on current impact. Hence, a higher level of $\alpha _1^*$ might be an indication that an organization is more focused on efficiently serving its beneficiaries right now than focusing on sustaining their operations. Hence, contrary to current perception that a high $\alpha _1^*$ always means that an organization uses the donations in an inefficient manner, it can be an indicator of responsible resource use.

The opposite is true for *b*, with increasing values describing less discounting of future donations, meaning that the NPO ascribes a higher value to future donation inflows. This fosters the second periods’ utility part, favoring less administrative expenses. $w_2$ does the same, but from a price effect viewpoint. Increases in $w_2$ account for higher deductions of utility from administrative expenses. If $w_2$ increases, decreases in $\alpha _1^*$ follow in response. This results in recovering donations in the future period, with the decrease in $\alpha _1^*$ continuing until the resulting utility from the donation surge in the future period cancels out the loss of utility from administrative efficiency of the first period.

Analyzing the partial derivatives of $\alpha _1^*$ with respect to $f_1$ shows the negative impact of fundraising expenses on administrative expenses. The analysis of the impact of $x_1$ reveals that the size of this effect is influenced both by the first period depending on $x_1$ and by the second period depending on *b*, $w_2$ and $p_2$, $g_2$ and $d_2$, the efficiency gain parameter $x_1$ has a strictly positive effect. Interestingly, the effect of $x_1$ is decreasing in $x_1$ itself.

**Fig. 2:**
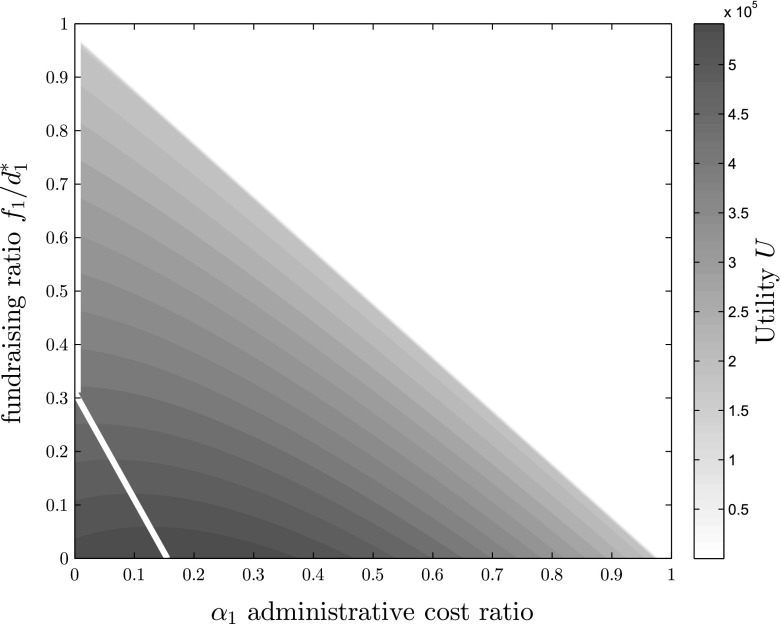
Utility level *U* depending on fundraising expenses $f_1$ and administrative cost ratio $\alpha _1$

While the price of giving and the program ratio include both administrative expenses as well as fundraising expenses in their effects, the efficiency gain considers only administrative expenses. How do the levels of fundraising and administrative expenses separately influence the resulting utility? In Fig. 2, the compound utility level is depicted depending on the values of administrative costs $\alpha _1$ and fundraising expenditure as $f_1$. Administrative cost levels $\alpha _1$ on the x-axis and the ratio of fundraising expenses relative to total budget $\frac{f_1}{d_1^*}$ on the y-axis result in varying levels of utility created, depicted by increasing intense shades of grey. Since $\alpha _1 +\frac{f_1}{d_1^*} \le 1$, the upper right triangle of the figure does not include any feasible solution, as this constraint is violated there. It can be seen that increasing levels of $\frac{f_1}{d_1^*}$ quickly decrease the optimal level of $\alpha _1$ towards 0, which is depicted by the white line for every possible value of $\frac{f_1}{d_1^*}$. In an environment of low fundraising expenses of $3\%$ of total donations, given the choice of parameters in this paper, $\alpha _1^* = 13.34\%$, while for $f_1=10 \%$, the ratio of $\alpha _1^*$ decreases to $9.84 \%$. Concluding, it can be said that NPOs cannot take decisions concerning administrative capacities separately from future impact considerations, but have to evaluate them jointly. Next we analyze how changes in parameters influence the optimal utility level $U^*$.

**Table 2:** Parameter’s partial derivatives with regard to $\alpha _1^*$

P	Partial derivative of $\alpha _1^*$ with respect to parameter P	Implication
*a*	$\frac{b \cdot w_2 \cdot d_2 \cdot g_2 \cdot p_2}{2 \cdot a^2 \cdot d_1^* \cdot x_1}$	+
*b*	$-\frac{(1-a) \cdot w_2 \cdot d_2 \cdot g_2 \cdot p_2 }{2 \cdot a \cdot d_1^* \cdot x_1}$	${-}$
$d_1^*$	$\frac{a \cdot f_1 \cdot x_1 - (a - 1) \cdot b \cdot d_2 \cdot g_2 \cdot p_2 \cdot w_2}{2 \cdot a \cdot d^2 \cdot x_1}$	+
$d_2$	$-\frac{(1-a) \cdot w_2 \cdot b \cdot g_2 \cdot p_2 }{2 \cdot a \cdot d_1^* \cdot x_1}$	${-}$
$f_1$	$\frac{-1}{2 \cdot d_1^*}$	${-}$
$g_2$	$-\frac{(1-a) \cdot w_2 \cdot d_2 \cdot b \cdot p_2 }{2 \cdot a \cdot d_1^* \cdot x_1}$	${-}$
$p_2$	$-\frac{(1-a) \cdot w_2 \cdot d_2 \cdot g_2 \cdot b }{2 \cdot a \cdot d_1^* \cdot x_1}$	${-}$
$w_2$	$-\frac{(1-a) \cdot b \cdot d_2 \cdot g_2 \cdot p_2 }{2 \cdot a \cdot d_1^* \cdot x_1}$	${-}$
$x_1$	$\frac{a\cdot d_1^* - (a-1) \cdot b \cdot d_2 \cdot g_2 \cdot p_2 \cdot w_2}{2 \cdot a \cdot d_1^* \cdot x_1^2}$	+

The partial derivatives for the parameters with respect to the optimal level of utility $U^*$ are summarized in Table 3, which can be found in the appendix. While the effect of future impact maximization has been investigated thoroughly in the literature (e.g. Steinberg (1986) regarding budget maximization), one very interesting question is the impact of efficiency gains on the optimal utility $U^*$.

#### Proposition 2

There exists a threshold $x_1 > \left( \frac{a \cdot d_1^* -(a-1) \cdot b \cdot d_2 \cdot g_2 \cdot p_2 \cdot w_2}{a \cdot (d_1^* -f_1)}\right) (\equiv t_1)$ above which the utility $U^*$ increases if the efficiency gain parameter $x_1$ increases.

As Table 2 indicates, an increase in *x* leads to an increase in $\alpha _1^*$. The increase in $\alpha _1^*$ leads to positive (efficiency gain) and negative (price effect and program ratio effects) on the utility $U^*$. If $x_1$ is below the threshold $t_1$, then the later effects dominate, leading to a negative effect on utility. Above the threshold, the reverse holds, as Proposition 2 describes. This shows that for an NPO, efficiency gains are not necessarily beneficial.

#### Corollary 1

The threshold $t_1$ of Proposition 2 is increased by the components of the future budget part of the utility function. The weight $1-a$ and the parameters *b*, $d_2$, $w_2$, $g_2$, $p_2$ and $f_1$ all reduce the threshold $t_1$.

Corollary 1 presents the counter-intuitive finding, that the effect of the efficiency gain of administrative costs (in the first period) on the optimal utility is determined by the parameters of the second period. At a second glance, the importance of the first period utility relative to the second period determines the increase in utility. Therefore, increasing administrative capacities is more useful for an NPO focusing on impact generation, than an NPO determined to increase future budget and impact.

#### Proposition 3

While the effect of the present donation parameter $d_1^*$ on the optimal level of administrative cost ratio $\alpha _1^*$ is positive, the second period donation parameter $d_2$ has a negative impact.

**Fig. 3:**
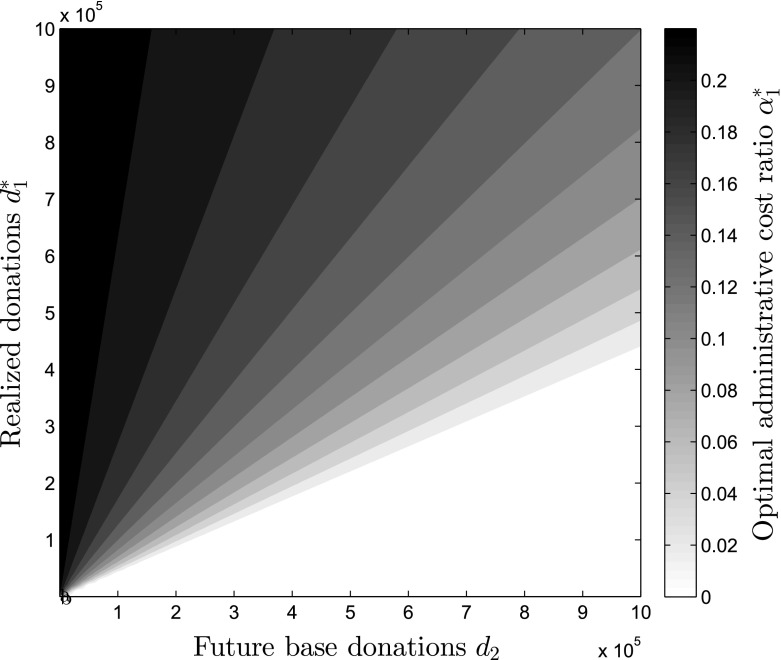
Optimal level of administrative cost ratio depending on donation levels $d_1^*$ and $d_2$

The different impact of the two periods’ donation parameters is stated by Proposition 3. The current donations $d_1^*$ increase the optimal level of $\alpha _1^*$ due to higher potential impact generated in the current period, which can be facilitated through higher administrative efficiency and, thus, higher administrative expense ratios. The future period contrasts this effect since it aims at maximizing the future impact only, leading to lower administrative cost ratios to decrease the price effect on donations. In Fig. 3, the impact of different levels of $d_1^*$ and $d_2$ on the optimal level of administrative cost ratio $\alpha _1^*$ can be seen. On the x-axis, the future donation potential $d_2$ is depicted, while on the y-axis the realized donations $d_1^*$ are represented. The resulting coloring describes the optimal level of administrative spending $\alpha _1^*$ for the combinations of these two donation parameters and helps to understand how $\alpha _1^*$ depends on the fluctuation of the donations between periods. If the future base donations are very low, it can be the case that a relatively high level of $\alpha _1^*$ is optimal. The opposite is true if the future base donations are high compared to the realized donations $d_1^*$.

#### Corollary 2

The parameters *b*, $w_2$, $g_2$ and $p_2$ increase the partial derivative of $\alpha _1^*$ with respect to $d_1^*$, and decrease the partial derivative of $\alpha _1^*$ with respect to $d_2$. The parameters $x_1$ and $d_1^*$ have the respective opposite effects.

An increase in $d_1^*$ leads to an increase in the administrative cost ratio $\alpha _1^*$, while an increase in $d_2$ leads to a decrease. The magnitude of both effects is increased by *b*, $w_2$, $g_2$ and $p_2$, and decreased by $d_1^*$ and $x_1$, for $d_2$ also by *a*. NPOs that are indifferent between current and future budget (i.e. $a=0.5$) will increase (decrease) $\alpha _1^*$ in a more severe manner as $d_1^*$ ($d_2^*$) increases. A similar trend is true for NPOs that face a highly competitive donation ’market’ (i.e., NPOs with a high $w_2$). This means, that the importance of changing donation levels for administrative cost ratios is especially relevant for organizations with a high discount factor, a high advertisement effect and a high price effect.

It can be stated that the effects of fluctuating donations and willingness to donate can even have detrimental effects on the administrative capacities. If future willingness to donate is expected to increase, the level of administrative expenses could be reduced from an NPO focused on sustaining operations by maximizing future impact. Contrary, NPOs are advised by the proposed model to increase administrative ratios if lower willingness to donate can be foreseen to make better use of the current budget.

### Influence of information transparency

Not only the exact ratio of administrative costs has an impact on the willingness to donate of private donors, but also the amount of information that donors have concerning the use of funds by the NPOs. In the previously discussed Model 1b, fully informed donors are investigated. There is a strong move towards increased availability of financial information. Various institutions, including ‘charity watchdogs’ like Guidestar or Charity Navigator, aim at increasing NPO financial transparency by increasing accessibility of NPO tax data. This data comes from the US Internal Revenue Service (IRS) and is well suited for the calculation of overhead cost ratios. This transparency is considered as an important factor in the non-profit starvation cycle (Lecy and Searing 2015). The important question is whether this strive for more transparency is beneficial to the various stakeholders.

Thus, the parameter $i_2$, introduced to measure this transparency as information availability, is used. $i_2$ can take any value between 0 and 1, with 0 describing a situation in which no information about the overhead ratios is available for donors, and 1 describing a situation, where every donor has all financial information available. Consequently, lower values of $i_2$ will raise the importance of the second periods’ impact utility measure since the impact section is unaltered by this parameter.

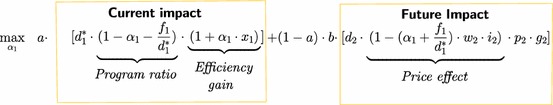



**Model 2:** Compound utility function including donor information parameter i2 (price effect)

subject to

5 $$\begin{aligned} \alpha _1 +\frac{f_{1}}{d_1^*}&\le 1 \end{aligned}$$

6 $$\begin{aligned} \alpha _1&\ge 0 \end{aligned}$$

The optimal level $\alpha _1^*$ and the respective utility $U^*$ are given by

7 $$\begin{aligned} \alpha _1^*&=\frac{1}{2}- \frac{1}{2 \cdot x_1}- \frac{f_1}{2 \cdot d_1^*} - \frac{(1- a) \cdot b \cdot i_2 \cdot w_2 \cdot d_2 \cdot g_2 \cdot p_2}{2 \cdot a \cdot d_1^* \cdot x_1} \nonumber \\ U^*&=\frac{a \cdot d_1^* - (a-1) \cdot b \cdot d_2 \cdot g_2 \cdot p_2 \cdot w_2 \cdot i_2)^2}{4 \cdot a \cdot d_1^* \cdot x_1} + \frac{a \cdot (d_1^* - f_1)^2 \cdot x_1}{4 \cdot d_1^*} \nonumber \\&\quad + \frac{a \cdot d_1^* \cdot (d_1^* -f_1) {-} 2 \cdot (a{-}1) \cdot b \cdot d_1^* \cdot d_2 \cdot g_2 \cdot p_2 {+} (a{-}1) \cdot b \cdot d_2 \cdot (d_1^* {+}f_1) \cdot g_2 \cdot p_2 \cdot w_2 \cdot i_2}{2 \cdot d_1^*} \end{aligned}$$

8 $$\begin{aligned} \frac{\partial \alpha _1^*}{\partial i_2}= -\frac{ (1-a) \cdot b \cdot w_2 \cdot d_2 \cdot g_2 \cdot p_2}{2 \cdot a \cdot d_1^* \cdot x_1} \end{aligned}$$

#### Proposition 4

Increasing the information available to private donors concerning financial data decreases the optimal level of the administrative cost ratio $\alpha _1^*$.

#### Corollary 3

While $x_1$, $d_1^*$ and *a* weaken this effect, *b*, $w_2$, $d_2$, $g_2$ and $p_2$ lead to an increased reduction of $\alpha _1^*$.

**Fig. 4:**
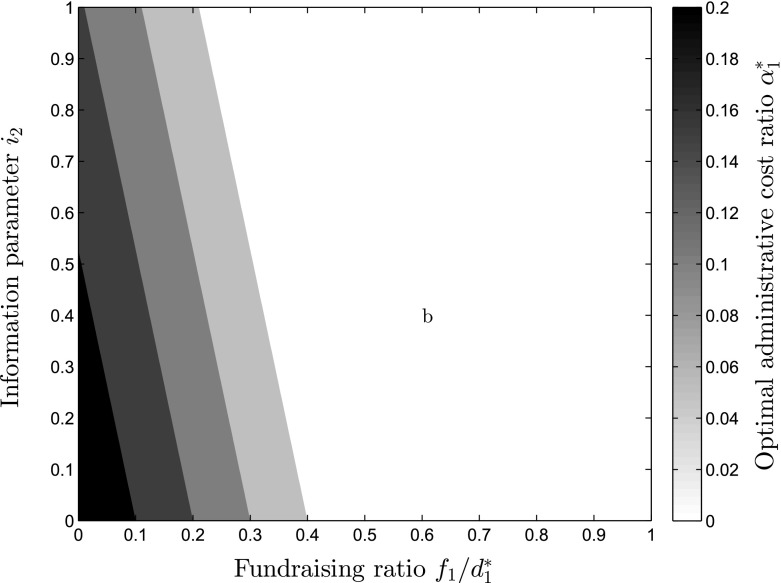
Optimal level of administrative cost ratio depending on fundraising expenses $f_1$ and information parameter $i_2$

Proposition 4 describes the impact of changes in the available financial information, lowering $\alpha _1^*$, as shown in Eq. 7. While this effect is especially strong in NPOs operating with high expected future base donations $d_2$, the effects are also stronger if *b*, the depreciation factor is higher, resulting in less depreciation. If the price of giving effect $w_2$ is high, the impact of information is also larger, while a higher efficiency gain factor $x_1$ can dampen this effect. Figure 4 exemplifies the complexity of this relationship of fundraising, information and optimal administrative cost levels. On the x-axis, the fundraising ratio relative to realized donations $\frac{f_1}{d_1^*}$ is represented, while the y-axis shows the level of information $i_2$. The coloring depicts the level of optimal administrative cost ratios $\alpha _1^*$ with darker shades denoting higher levels of $\alpha _1^*$. As can be seen, for low values of $i_2$, a relatively larger value of $\alpha _1^*$ is optimal even if $f_1$ takes a relatively high value, which can be attributed to the lack of information about overhead cost ratios. Contrarily, if $i_2$ rises, this is especially detrimental for NPOs with high levels of $f_1$. Additionally, it has to be stated, that this effect occurs only for increased *financial* information available and does not explore the effects of other performance measurement information on donation behavior.

**Fig. 5:**
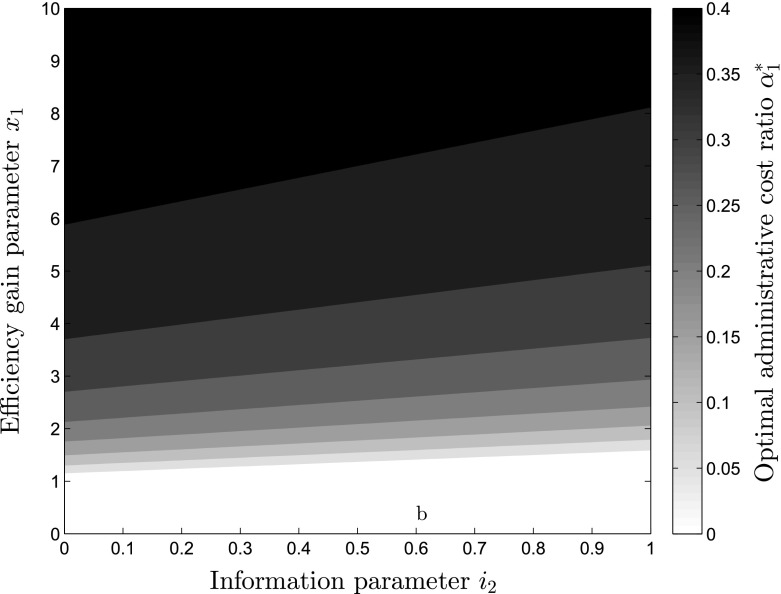
Optimal level of administrative cost ratio depending on the efficiency gain parameter $x_1$ and information parameter $i_2$

**Fig. 6:**
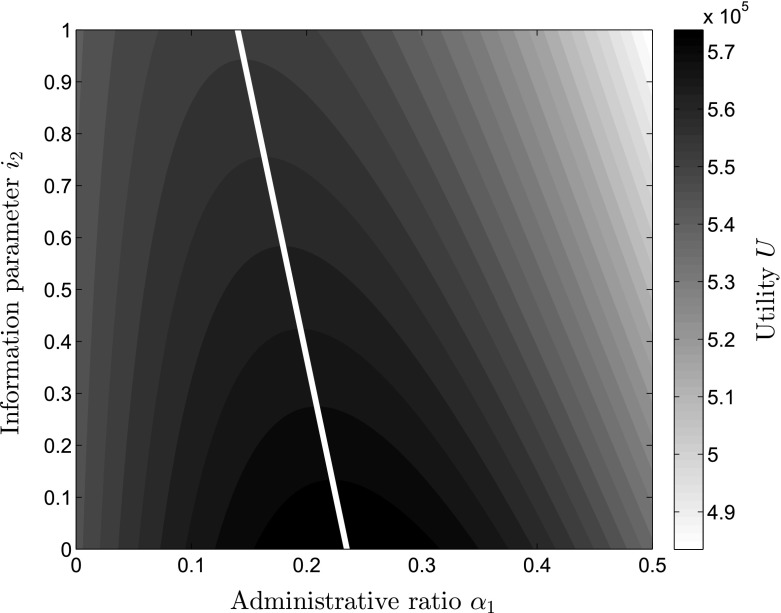
Utility level *U*, depending on administrative expense ratio $\alpha _1$ and information parameter $i_2$

Another interesting relationship exists between the size of the efficiency gain parameter $x_1$ and the level of information $i_2$ available to the donors. For very low $x_1<1$, $\alpha _1^*$ should always be 0 as $\alpha _1^*$ becomes negative, while with increases in $x_1$ the increases in the level of $\alpha _1^*$ occur fast but decrease in their speed, as can be seen in Fig. 5. On the x-axis, the level of information $i_2$ is depicted, while the efficiency gain parameter can be seen on the y-axis. The darker shades describe higher levels of optimal administrative cost ratios $\alpha _1^*$. Figure 5 helps understanding the high importance of the efficiency gain parameter $x_1$ for the level of $\alpha _1^*$. With increases in $i_2$, $\alpha _1^*$ is reduced, which becomes even more clear in Fig. 6. Here, different levels of utility are depicted depending on the information $i_2$ available to the donors on the y-axis and the administrative expense level $\alpha _1$ on the x-axis, exhibiting that through increasing the level of information, the level of $\alpha _1$ providing the highest possible utility is continuously shifted towards lower levels of $\alpha _1$ as can be seen from the white line depicting the optimal levels of $\alpha _1^*$ for each level of $i_2$.

Effects on $U^*$ will be described in the following. The partial derivative for *i* with respect to the utility function is given by:

9 $$\begin{aligned} \frac{(a{-}1) \cdot b \cdot d_2 \cdot g_2 \cdot p_2 \cdot ((a-1) \cdot b \cdot d_2 \cdot g_2 \cdot p_2 \cdot w_2 \cdot i_2 {-}a \cdot d_1^*{+}a \cdot x_1 \cdot (d_1^*{+}f_1))}{2 \cdot a \cdot d_1^* \cdot x_1} \end{aligned}$$

#### Corollary 4

Increases in the parameters $f_1$, $x_1$, and *a* all facilitate the negative impact of $i_2$, while *b*, $d_2$, $g_2$, $p_2$ and $w_2$ reduce the negative impact.

Concluding it can be stated that high transparency of financial data is especially detrimental to NPOs with a high potential for efficiency gains through increased administrative spending $x_1$. Of course, more knowledge about non-program usage of funds is especially bad for NPOs facing a donation ’market’ with low donation elasticities of price of giving, which is closely linked to $w_2$ and also NPOs which value future donations much less compared to current (*b* far less than 1).

## Conclusion

In this paper we developed a quantitative model to analyze the impact of fund allocations with the focus on administrative cost ratios. Based on the analytical models studied, an optimal level of administrative cost ratio has been determined. This ratio is bounded by 0.5.

The impact of different factors on the optimal level of administrative cost ratio has been at the center of the research. The optimal level is always increasing in the efficiency gain of increased administrative capacities, the associated utility created can only increase if the efficiency gain is above threshold $t_1$. Our results suggest that challenges posed by fluctuating donations can result in negative impact on administrative capacities in case of increased future willingness to donate. Thus, if NPOs anticipate a high future donation potential, their current impact receives less administrative support than optimal under a constant flow of donations. This highlights the importance of a steady flow of donations not only from an operational perspective in general but from the perspective of efficient administration of NPOs. This result is consistent with information received in a personal interview with The World Food Program (WFP). The WFP is struggling with highly fluctuating donations, which makes it difficult for them to make a sustainable plan for building relief infrastructures.

Furthermore, our research shows that the focus on financial performance measurements in NPOs, supported by increasingly available overhead cost information to donors, can lead to detrimental effects on utility created through resulting reduced administration cost ratios. This is especially true for NPOs facing high efficiency gains in increased administrative expenditure in a donation ’market’ with low donation elasticities of price of giving.

While the focus of this paper has been on the establishment of an analytic model for issues related to overhead and especially administrative cost ratios of NPOs, the properties of the model lend themselves to useful extensions and new research questions. Some foreseeable future research opportunities relate to the notion of competition in NPO operations by establishing a multi-NPO, multi-period setting suited to model the nonprofit starvation cycle. Another promising research direction is taking the size of an NPO into account when determining the efficiency gains, as small NPOs often require low administrative expenses, as much work can be done on a voluntary basis. When NPOs grow, however, the administrative requirements tend to grow even stronger (Lecy and Searing 2015). Further worthwhile research opportunities include the quality of the performance measurement and its impact on this framework. Furthermore, as has been shown in Sect. 3.1, the close relationship in decision-making between setting the expenses of fundraising $f_1$ and subsequently determining the use of the received donations (deciding on $\alpha _1$) should be investigated in an integrated way, providing future research opportunities beyond the scope of this paper. Further potential for research can be found in the efficiency gain function, which is linear in this research but could take different functional forms, and in introducing uncertainty for future base donations into account.

## Appendix

### Proofs

#### Proof of Proposition 1

Setting $f_1=0$ in Eq. 3 and using the assumptions of Table 1 (all variables non-negative and $a>0$), $\lim \limits _{x_1 \rightarrow \infty } \alpha _1^* = 0.5$.

#### Proof of Proposition 2

$$\begin{aligned} \frac{\partial U^*}{\partial x_1}&= \frac{-(a \cdot d_1^* - (a {-} 1) \cdot b \cdot d_2 \cdot g_2 \cdot p_2 \cdot w_2)^2 {+} a^2 \cdot (d_1^* {-} f_1)^2 \cdot x^2)}{4 \cdot a \cdot d_1^* \cdot x_1^2}&\text {is positive in case of} \\&(a \cdot d_1^* {-} (a {-}1) \cdot b \cdot d_2 \cdot g_2 \cdot p_2 \cdot w_2)^2 {<} a^2 \cdot (d_1^* {-}f_1)^2 \cdot x_1^2&\text { which can be reformulated:} \\ x_1&> \left( \frac{a \cdot d_1^* -(a-1) \cdot b \cdot d_2 \cdot g_2 \cdot p_2 \cdot w_2}{a \cdot (d_1^* -f_1)}\right) \end{aligned}$$

#### Proof of Corollary 1

Partial derivatives for the threshold $t_1$ of Proposition 2 with respect to the parameters *b*, $d_2$, $w_2$, $g_2$, $p_2$ and $f_1$ lead to the result that all these parameters have a negative impact on the threshold $t_1$.

#### Proof of Proposition 3

The partial derivatives of the optimal level of administrative expenses with respect to the two donation parameters $d_1^*$ and $d_2$ can be investigated under the assumptions stated in Table 1 requiring the parameters and variables to take on positive values. As $1-a >0$, all components of the first partial derivative are positive, leading to a total positive impact of donations, while for the partial derivative with respect to $d_2$, the minus sign reverses this effect.

$$\begin{aligned} \frac{\partial \alpha _1^*}{\partial d_1^*}&= \frac{a \cdot f_1 \cdot x_1 - (a - 1) \cdot b \cdot d_2 \cdot g_2 \cdot p_2 \cdot w_2}{2 \cdot a \cdot d_1^{*2} \cdot x_1} \\ \frac{\partial \alpha _1^*}{\partial d_2}&= -\frac{(1-a) \cdot w_2 \cdot b \cdot g_2 \cdot p_2 }{2 \cdot a \cdot d_1^* \cdot x_1} \end{aligned}$$

#### Proof of Corollary 2

Using the non-negativity assumptions of Table 1, the proof follows from

$$\begin{aligned} \frac{\partial \alpha _1^*}{\partial d_1^*}&= \frac{a \cdot f_1 \cdot x_1 - (a - 1) \cdot b \cdot d_2 \cdot g_2 \cdot p_2 \cdot w_2}{2 \cdot a \cdot d_{1}^{*2} \cdot x_1} \\ \frac{\partial \alpha _1^*}{\partial d_2}&= -\frac{(1-a) \cdot w_2 \cdot b \cdot g_2 \cdot p_2}{2 \cdot a \cdot d_1^* \cdot x_1} \end{aligned}$$

Partial derivatives with respect to the mentioned parameters show the described impact of the parameters.

#### Proof of Proposition 4

Using the non-negativity assumptions of Table 1, the proof follows from

$$\begin{aligned} \frac{\partial \alpha _1^*}{\partial i_2} = -\frac{ (1-a) \cdot b \cdot w_2 \cdot d_2 \cdot g_2 \cdot p_2}{2 \cdot a \cdot d_1^* \cdot x_1} \end{aligned}$$

#### Proof of Corollary 3

Using the non-negativity assumptions of Table 1, the proof follows from

$$\begin{aligned} \frac{\partial \alpha _1^*}{\partial i_2} = -\frac{ (1-a) \cdot b \cdot w_2 \cdot d_2 \cdot g_2 \cdot p_2}{2 \cdot a \cdot d_1^* \cdot x_1} \end{aligned}$$

Partial derivatives with respect to the mentioned parameters show the described impact of the parameters.

#### Proof of Corollary 4

Partial derivatives for the partial derivative of $i_2$ with respect to $U^*$ with respect to the parameters *a*, $f_1$ and $x_1$ lead to the result that all these parameters facilitate the negative impact of $i_2$ on $U^*$, while *b*, $d_2$, $g_2$, $p_2$ and $w_2$ decrease this impact.

### Utility at optimal administrative cost ratio level $\alpha _1^*$

See Table 3.

**Table 3:** Parameter’s partial derivatives with respect to $U^*$

P	Partial derivative of $U^*$ with respect to parameter P
*a*	$\frac{-b^2 \cdot d_2^2 \cdot g_2^2 \cdot p_2^2 \cdot w_2^2+a^2 \cdot (2 \cdot b \cdot d_1^* \cdot d_2 \cdot g_2 \cdot p_2 \cdot (w_2 \cdot (x_1 -1)-2 \cdot x_1)-2 \cdot d_1^* \cdot f_1 \cdot x_1 \cdot (1+x_1 )+d_1^{*2} \cdot (1+x_1)^2+(b \cdot d_2 \cdot g_2 \cdot p_2 \cdot w_2+f_1 \cdot x_1)^2)}{4 \cdot a^2 \cdot d_1^* \cdot x_1}$
	positive impact if $a> \frac{b \cdot d_2 \cdot g_2 \cdot p_2 \cdot w_2}{\sqrt{2 \cdot b \cdot d_1^* \cdot d_2 \cdot g_2 \cdot p_2 \cdot (w_2 \cdot (x_1 -1)-2 \cdot x_1)-2 \cdot d_1^* \cdot f_1 \cdot x_1 (1+x_1)+d_1^{*2} \cdot (1+x_1)^2+(b \cdot d_2 \cdot g_2 \cdot p_2 \cdot w_2+f_1 \cdot x_1)^2}}$
$f_1$	$\frac{-b \cdot d_2 \cdot g_2 \cdot p_2 \cdot w_2 +a \cdot (b \cdot d_2 \cdot g_2 \cdot p_2 \cdot w_2 +f_1 \cdot x_1 -d_1^* \cdot (1+x_1))}{2 \cdot d_1^*}$
	negative impact if $f_1 < d_1^* + \frac{d_1^*}{x_1} + \frac{(1-a) \cdot b \cdot d_2 \cdot g_2 \cdot p_2 \cdot w_2}{a \cdot x_1}$
$x_1$	$\frac{-(a \cdot d_1^* - (a - 1) \cdot b \cdot d_2 \cdot g_2 \cdot p_2 \cdot w_2)^2 + a^2 \cdot (d_1^* - f_1)^2 \cdot x_1^2)}{4 \cdot a \cdot d_1^* \cdot x_1^2}$, positive impact if $x_1 > \left( \frac{a \cdot d_1^* -(a-1) \cdot b \cdot d_2 \cdot g_2 \cdot p_2 \cdot w_2}{a \cdot (d_1^* -f_1)}\right)$
$w_2$	$\frac{(a-1) \cdot b \cdot d_2 \cdot g_2 \cdot p_2 \cdot ((a-1) \cdot b \cdot d_2 \cdot g_2 \cdot p_2 \cdot w_2 -a \cdot d_1^*+a \cdot x_1 \cdot (d_1^* +f_1))}{2 \cdot a \cdot d_1^* \cdot x_1}$, negative impact if $w_2< \frac{a \cdot d_1^* \cdot x_1 +a \cdot f_1 \cdot x_1 -a \cdot d_1^*}{(1-a) \cdot b \cdot d_2 \cdot g_2 \cdot p_2}$
$i_2$	$\frac{(a-1) \cdot b \cdot d_2 \cdot g_2 \cdot p_2 \cdot ((a-1) \cdot b \cdot d_2 \cdot g_2 \cdot p_2 \cdot w_2 \cdot i_2 -a \cdot d_1^* +a \cdot x_1 \cdot (d_1^* +f_1))}{2 \cdot a \cdot d_1^* \cdot x_1}$, negative impact if $i_2< \frac{a \cdot d_1^* \cdot x_1+a \cdot f_1 \cdot x_1-a \cdot d_1^*}{(1-a) \cdot b \cdot d_2 \cdot g_2 \cdot p_2 \cdot w_2}$

## Footnotes

1 The impact of the advertisement effect can be approximately explored in our model by changing the parameter $f_1$ and $d_1^*$ simultaneously. However, this analysis is out of scope of our research.
